# Ethylene Glycol Poisoning: A Case Study of a 71-Year-Old Male with a History of Alcohol Abuse and Suicide Attempts

**DOI:** 10.7759/cureus.57850

**Published:** 2024-04-08

**Authors:** Mohammad Q Jafri, Amardeep Parhar, Matthew Frank, Ivan Nikiforov

**Affiliations:** 1 Internal Medicine, Ocean University Medical Center, Brick Township, USA; 2 Pulmonary and Critical Care, Ocean University Medical Center, Brick Township, USA; 3 Internal Medicine/Pulmonary and Critical Care, Donald and Barbara Zucker School of Medicine at Hofstra/Northwell, Hempstead, USA

**Keywords:** ethylene glycol poisoning, alcohol poisoning, high anion gap metabolic acidosis, ethanol, fomepizole, osmolar gap, ethylene glycol

## Abstract

A 71-year-old male with a history of alcohol abuse and multiple suicide attempts was brought to the emergency department in an unconscious state. Initial assessment revealed profound obtundation and malnutrition. Laboratory findings demonstrated a significant anion gap metabolic acidosis with a high osmolar gap, suggestive of possible toxic alcohol ingestion. Despite negative serum alcohol levels, ethylene glycol poisoning was confirmed with a level of 226. Treatment included fluid resuscitation, bicarbonate therapy, and fomepizole administration. However, due to progressive multi-organ failure, continuous veno-venous hemodialysis was initiated. Despite interventions, the patient deteriorated rapidly, leading to a decision for hospice care, ultimately resulting in death. Ethylene glycol poisoning presents significant challenges in management, with potential complications including renal failure and multi-organ dysfunction. Fomepizole remains the cornerstone of treatment, but additional therapies such as ethanol administration were considered but ultimately deemed unnecessary due to associated risks. This case highlights the complexity and severity of ethylene glycol poisoning, emphasizing the need for early recognition and aggressive management strategies.

## Introduction

Ethylene glycol (C2H6O2) is a hazardous alcohol present in various household and industrial products [1]. It is a colorless, sweet-tasting liquid commonly found in antifreeze but is occasionally used in other applications, such as industrial solvents. In cases of deliberate exposure, it may be motivated by suicide attempts or a desire for intoxication in the absence of ethanol [1].

According to data from the American Association of Poison Control Centers (AAPCC), over 5,000 exposures are reported annually in the United States [2]. Exposure to this alcohol can be extremely perilous and it needs quick recognition by the respective clinicians in order to improve patient outcomes. Managing ethylene glycol exposure involves supportive care, close laboratory monitoring, and the use of antidote therapies such as ethanol or fomepizole, and occasionally dialysis [1].

We present the case of a 71-year-old male who was discovered unconscious in a hotel room, prompting his admission to the emergency department via emergency medical services.

## Case presentation

A 71-year-old male was brought to the emergency department via emergency medical services after being found unconscious in a hotel room. Past medical history was significant for alcohol abuse and multiple suicide attempts. The patient was living in a hotel room, where he was found by hotel staff covered in feces and urine with empty bottles of alcohol. It was unknown how long the patient may have been on the ground unconscious. Of note, he had about 10 visits to the ED in the last year for alcohol intoxication and a few different admissions for suicidal ideations. 

On presentation, blood pressure was 78/40 mmHg, pulse was 106 beats per minute, and other vital signs were within normal limits (Table 1). On physical examination, the patient appeared malnourished and obtunded, only responding to painful stimuli. The initial metabolic panel was significant for anion gap of 28 mmol/L, bicarbonate of 8.0 mmol/L, sodium 156 mmol/L, blood urea nitrogen 13 mg/dL, glucose 140 mg/dL, lactic acid >20 mmol/L, serum osmolality 450 mOsm/kg, and osmolar gap 126 mOsm/kg (Table 2). The drug screen was negative and blood alcohol <0.0005%. Urinalysis was unremarkable. Arterial blood gas (ABG) obtained several hours after presentation, while the patient was on bilevel positive airway pressure (BiPAP) with settings of 16/6, revealed a pH of 6.50, carbon dioxide 19.7, oxygen 155.9 with a bicarbonate level 1.5 (Table 3). An electrocardiogram showed sinus tachycardia with a prolonged QTc of 528 ms. Chest x-ray and computed tomography head without contrast both showed no acute pathology.

**Table 1:** Initial set of vitals on presentation to the emergency department

Vitals	Patient Values
Temperature	99.3 °F
Pulse	106 beats per minute
Blood Pressure	78/40 mmHg
Respiratory Rate	19 breaths per minute
Weight	73 kg (160 Ib)
Height	177 cm (5"10')

**Table 2:** Initial comprehensive metabolic panel including serum osmolality and osmolar gap values

Lab Test	Value	Reference Range
Glucose	140	70 - 99 mg/dL
Sodium	156	136 - 145 mmol/L
Blood Urea Nitrogen	13	5 - 25 mg/dL
Creatinine	0.83	0.61 - 1.24 mg/dL
Bicarbonate	8	24 - 31 mmol/L
Anion Gap	28	5 - 15 mmol/L
Lactic Acid	>20	0.5 - 1.9 mmol/L
Serum Osmolality	450	280 - 300 mOsm/kg
Osmolar Gap	126	<10 mOsm/kg water

**Table 3:** Arterial blood gases obtained after several hours of BiPAP with settings of IPAP of 16 and EPAP of 6 (16/6) BiPAP: bilevel positive airway pressure ventilation; IPAP: inspiratory positive airway pressure; EPAP: expiratory positive airway pressure

Arterial Blood Gas	Value	Reference Range
pH	6.50	7.35 - 7.45
Carbon Dioxide	19.7	35.0 - 45.0 mmHg
Arterial Oxygen	155.9	75.0 - 100.0 mmHg
Bicarbonate	1.5	22.0 - 28.0 mmol/L

Given the significant metabolic acidosis along with the high osmolar and anion gap and prior history of suicidal behaviors, poisoning by either ethylene glycol, alcohol ketoacidosis, or methanol was strongly considered as a part of the initial differential diagnosis. The patient was intubated and sedated in the ED. He then received 4L boluses of lactated ringers (LR) along with being started on 500 mEq bicarbonate, dextrose 5% in water (D5W) 100 mEq at 200 cc/hr. One dose of vancomycin 1000 mg and one dose of fomepizole 1000 mg were also administered in the ED. A few hours following the administration of these medications, the patient's blood pressure improved to 110/56 mmHg but he remained obtunded and was thus transferred to the intensive care unit.

## Discussion

As mentioned, the significant metabolic acidosis, high osmolar and anion gap, and prior history of suicidal behaviors, poisoning by either ethylene glycol, alcohol ketoacidosis, or methanol was a part of the initial differential diagnosis. To investigate these toxicities, methanol, ethanol, and ethylene glycol levels were collected and sent. Ethylene glycol levels eventually returned positive with a level of 226, while methanol and ethanol levels were negative.

Despite the fomepizole administration being started early and being continued, the patient deteriorated and required continuous venovenous hemodialysis one day following admission. This was anticipated because ethylene glycol metabolites are metabolized by the kidney and lead to reversible acute kidney injury (AKI). This slows the elimination of ethylene glycol creating a feedback loop leading to further renal failure due to the buildup of these damaging metabolites. Tubule obstruction from precipitated oxalate crystals may also contribute [3]. The accumulation of ethylene glycol metabolites leads to a significant anion gap metabolic acidosis [3]. This was seen in the patient with an initial pH of 6.50, absent reflexes, and CNS depression. Hemodialysis plays a crucial role in managing severely poisoned patients and stands as the most effective approach for swiftly eliminating both toxic acid metabolites and the parent alcohol. It should also be continued until the blood pH is normal and ethylene glycol levels are < 3.2 mmol/L [4].

Fomepizole is the first-line treatment for both methanol and ethylene glycol poisoning. When administered, fomepizole should be continued until the diagnosis of toxic alcohol ingestion has been ruled out or until blood pH is normal and serum alcohol concentration is < 20 mg/dL in the presence of either retinal or renal injury [4].

There were discussions about adding ethanol to the fomepizole treatments of the current patient; however, it was ultimately decided against. Both fomepizole and ethanol therapy effectively lower methanol and ethylene glycol levels; however, recent studies have revealed that there is no benefit to adding ethanol therapy to fomepizole therapy in methanol- and ethylene glycol-poisoned patients [4]. Although, ethanol may be effective at lowering methanol and ethylene glycol levels [5,6], administering intravenous (IV) ethanol presents challenges due to the complexities of dosing and maintaining optimal levels. Hospital pharmacies must compound it, and its infusion can irritate veins while potentially affecting fluid balance in patients with reduced urine output. The sedative and behavioral effects of ethanol are particularly limiting, leading to potential obtundation and an increased risk of complications such as aspiration [7].

The patient proceeded to receive a few days of continuous venovenous hemodialysis, which resulted in improved acidosis; however, he eventually became oliguric. He also continued to receive fomepizole with his repeat ethylene glycol levels on subsequent days showing a downtrend. Despite these interventions, he developed multi-organ failure and rapidly declined to the point where his family decided in favor of hospice care where the patient expired shortly after.

## Conclusions

This case highlights the severity and potential lethality of ethylene glycol poisoning, particularly evidenced by the development of severe metabolic acidosis with a pH of 6.50. It is imperative for healthcare professionals to maintain a high index of suspicion for toxic alcohol ingestions in patients presenting with unexplained acid-base derangements. Prompt recognition, early initiation of specific antidotal therapy such as fomepizole or ethanol, and aggressive supportive care remain the most crucial determinant to patient outcomes and reducing morbidity and mortality.
